# Construction of teaching system of public art major using CMOS image sensor technology

**DOI:** 10.1038/s41598-024-56224-w

**Published:** 2024-03-06

**Authors:** Xueyin Yang

**Affiliations:** School of Art and Design, Yellow River Conservancy Technical Institute, Kaifeng, 475000 China

**Keywords:** Teaching of public art, Diversified public art perspectives, CMOS image sensor technology, Teaching system construction, Computational science, Computer science, Scientific data

## Abstract

The traditional public art education model has many drawbacks. After all, this teaching model is the most common teaching model. Most colleges and universities still rely on the traditional teaching mode. This teaching mode is not attractive, boring, and low cost, so the popularity rate is high. The digital interactive design with multimedia courseware as the main body has played a great role in promoting the teaching of public art education in colleges and universities. In this paper, when teachers use multimedia courseware for teaching, because the computer cannot process physical signals, part of the hardware is a converter that converts physical signals such as light and pictures received by the CMOS sensor into digital signals and inputs them to digital signals and analog signals. Through the converter, the two-way transmission of teacher and student information in the teaching process of public art majors from the perspective of diversified public art was realized. At the same time, the information elements of sound, image and text were integrated into the interactive works of public art majors from the perspective of diverse public art, so as to stimulate students’ interest in public art learning. Combined with the application of CMOS image sensor technology in diversified public art teaching, a questionnaire survey was conducted to explore the satisfaction of students in public art education and teaching of colleges and universities by CMOS image sensor technology, 50% of the students agreed with the proposal in this article. In this paper, the diversified public art teaching based on CMOS image sensor technology was derived outside the classroom, and the teaching was related to students’ life, so as to improve the artistic atmosphere of the campus and truly improve the teaching effect of public art education in colleges and universities.

## Introduction

China has a vast territory and a large population, and different places have different artistic and cultural characteristics, which are very valuable artistic resources. The public art administrators of colleges and universities should pay attention to and explore the local art and cultural resources. Combining with the actual situation of the school and adapting measures to local conditions, conditions have been created to solve the difficulties in public art education, so that the public art education in Chinese universities can not only be fully developed, but also have unique regional characteristics^1^. All colleges and universities should cooperate and coordinate in many aspects and links according to their own characteristics. They also jointly explore the school art education management mode suitable for each college’s own situation and continuously promote the improvement of the quality of art education and teaching in colleges and universities, as well as cultivate all-round development of college students. Therefore, it is necessary to conduct in-depth analysis and research on the public art education curriculum system. The contradictions and problems in public art education are comprehensively and objectively recognized, and countermeasures are explored to build a more ideal public art curriculum system and implement art education more effectively.

Public art courses offered in colleges and universities focus on exploring different artistic practices that relate to public spaces. These courses cover various forms of art, including sculptures, murals, installations, and performance art, and emphasize their impact on public spaces. Students who enroll in these courses investigate the intersection of art and the public sphere, exploring concepts related to civic engagement, cultural expression, and community dialogue. The primary objective of these courses is to equip students with the skills and knowledge to create art that not only reflects the diverse voices within a community but also plays an active role in shaping the cultural and social fabric of public spaces. Zheng^2^ examined urban sculpture production to understand how a public art scene is generated in China. Darivemula et al.^3^ believed that public art projects directly addressed the problem of physician burnout. Caldarola^4^ believed that some of NGPA’s works should be based on their artistic features, rather than contrasted with conceptual art works. Macarow et al.^5^ has created, promoted and documented a range of socially engaged arts and innovative medical practices in his exploration of end-of-life in a Stockholm nursing home. Zagkotas and Fykaris^6^ formed a teaching program based on Harvard University’s “Artistic Thinking Project”. However, the teaching method of public art majors proposed by them is not very effective. This paper introduces CMOS image sensor technology to optimize it.

CMOS imaging techniques are widely used in teaching systems due to their advanced technology and cost-effectiveness in capturing visual information. The basic structure of a CMOS sensor consists of an array of pixels, each having a photodiode that converts incident light into electrical signals. These electrical signals are then processed through a readout circuit, with color filtering using Bayer filters to capture diverse color information. Technical considerations such as optimizing the signal-to-noise ratio, dynamic range, pixel size, and resolution are essential to ensure high-quality image output. The low power consumption of CMOS technology is advantageous in teaching systems, as it aligns with the need for energy-efficient and portable solutions. The incorporation of on-chip image processing capabilities further enhances the versatility of CMOS sensors, allowing for features like white balance adjustment and exposure control. The continuous advancements in CMOS technology, including innovations such as back-illuminated sensors, have contributed to their widespread adoption in educational settings, facilitating visual demonstrations, image analysis, and various computer vision applications. 

CMOS image sensor technology has a strong sense of design, which makes students focus on the courseware. Nie et al.^7^ proposed a fast multiple correlation sampling technique based on Successive Approximation Register Analog-to-digital Converters (SAR ADCs) for low noise CMOS image sensors. Marcelot et al.^8^ proposed and detailed a thermionic model that varied with exponential implantation. Stefanov et al.^9^ developed a new pixel design scheme based on CMOS image sensor technology. Goiffon et al.^10^ studied the Total Ionizing Dose (TID) effect of a CMOS Image Sensor (CIS) based demonstrator for ITER remoting. Fei and Theuwissen^11^ focused on the effect of the operating temperature of CMOS Image Sensors (CIS) on pixel performance. However, their proposed CMOS image sensor technology is rarely studied in the teaching field.

This paper used CMOS image sensor technology to study the content of public art courses offered by some colleges and universities, and summarized the common points and differences to find out the shortcomings of the public art design curriculum in colleges and universities, in order to look forward to better cultivate design talents that conform to the development of the times. Then, under this framework, it deeply analyzed the content, composition and function characteristics of the implementation mechanism of art education and found out the problems existing in the existing implementation mechanism, as well as analyzed the reasons. The use of multiple exposure techniques to extend the dynamic range inevitably increased the pixel readout rate. The compact readout scheme can greatly improve the readout rate problem brought by the multiple exposure technology only by improving the exposure and readout timing, without adding any additional consumption to the CMOS image sensor. This paper took the public art major curriculum system in colleges and universities as the specific research object. Whether in the area of public art education research or in the method system of public art education research, it is the perfection of the whole public art education research system. Only 3 universities teach public art courses, accounting for more than 0.15% of the total number of undergraduates.

## Application of CMOS image sensor technology in the teaching system of public art majors

### Diversified teaching system for public art majors

**Figure 1:**
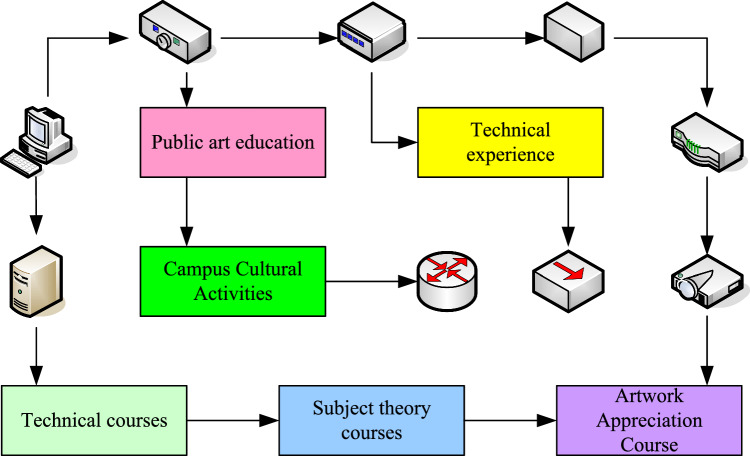
Categories of public art courses.

Culture is a peculiar phenomenon of human society. While creating different cultures, human beings also exist in a certain cultural background. Art is the most important carrier of culture. Therefore, the postmodern view of art education emphasizes the respect for multiculturalism in art education, and believes that art education must maintain the unique culture of the nation and accept the multiculturalism of the world. This actually shows that the selection of art curriculum content should solve the problem of coordinating other country's art with the content of the national art. The adjustment and optimization of the public art curriculum structure is to change the current situation of single curriculum structure, unclear level and subject-based in the past, and to re-examine the relationship between curriculum levels and the unique value of art subject courses and comprehensive courses in different fields to students' development. However, the current public art curriculum system or too much attention to theoretical courses is not satisfactory in terms of connecting with real life and cultivating practical ability. This requires that the relationship between theoretical courses and practical courses must be handled well. It is necessary to ensure the establishment of necessary courses of aesthetic principles and art appreciation, and to appropriately strengthen courses of artistic experience and practical content^12,13^. The categories of public art courses are shown in Fig. 1.

1. *The pertinence of public art curriculum goals* since art education itself pursues the full development of personality and the perfection of personality, it is very important to clarify the needs of college students in the formulation of public art curriculum goals^14^.
2. *Systematization of public art curriculum goals* at present, the level of attention to students' needs in the design of public art curriculum goals in Chinese universities is not in place. The art courses in many colleges and universities fail to make a reasonable assessment of the actual situation of the students before they are opened. Most courses face many students at the same time, and they are required to carry out art education with a unified training goal, resulting in a series of problems such as scattered and unsystematic knowledge acquired by students and unsatisfactory teaching effect. Therefore, in the design of public art curriculum objectives, the differences and levels of students’ needs are considered. The corresponding curriculum objectives are designed respectively, and the continuity of the objectives is maintained to ensure the continuity of art education, so that students can really benefit from their learning. At the same time, it can also avoid the indiscriminate chaotic state of different levels of art education and cause more waste of resources. The functional expression of the output state is^15^:
  1 $$ Q^{N + 1} = f\left( {x,Q^{N} } \right) $$
3. *Scientization of the compilation of public art curriculum objectives* the establishment of public art curriculum objectives is to point out the direction for public art education. It also makes the teaching behavior of art orderly and meaningful, and avoids blindness, which requires the design of curriculum goals to focus on scientific. The scientific nature is that the determination of the goal should be specific and clear, and it is easy to operate and grasp. That is to say, the expression of the objectives of the public art curriculum should take into account the requirements of both quality and quantity. If there is only the requirement of quality, the goal would appear abstract and not concrete; if there is only the requirement of quantity, the goal would be mere formality without substance. At present, there are many problems in the goal design of public art courses in colleges and universities. That is, it pays more attention to the quality requirements, but does not give certain attention to the quantitative requirements. It also leads to the generalization and abstraction of the target expression of public art courses, and the lack of operability in teaching^16^.
  On the other hand, the public art curriculum goals also involve the content of the emotional field. That is to say, the goal of emotional learning occupies a very important position in art education, but it is very difficult to write observable and measurable curriculum goals for learning in the emotional field. This kind of goal is aimed at emotional learning, which requires clearly specifying the activities and situations that learners should participate in, but does not propose a measurable learning structure. Although there is no precise specification of what should be learned from teaching activities, it is helpful to focus on the content of emotional teaching in public art courses. In general, the scientific goal design of public art curriculum is to deal with the problem of abstraction of the previous goal expression, and the second is to consider all fields of study^1^.
4. *Individualization of the objectives of public art courses* different universities reflect their unique uniqueness and development laws as a whole due to their own characteristics, subject settings, and professional settings, and the design of the target system of public art courses should also reflect this difference. In a word, in the formulation of public art curriculum objectives, both the characteristics of the university itself and the characteristics of the curriculum itself should be taken into account, fully reflecting the individualized characteristics of public art curriculum objectives. The total readout time is at most the same as the total frame cycle time, so the maximum value of the pixel readout time can be expressed as^17^:
  2 $$ T_{total} = \frac{{T_{F} }}{R} $$

In the management of public art education, managers should gradually pursue the effect of public art education management in colleges and universities, and should not be hasty. The purpose of public art education management is to enhance and expand the function of public art education, and it is necessary to pursue the effect of art management. However, many management mechanisms cannot be blindly established to pursue higher effects, because this would increase the burden. Haste is not enough. This not only fails to achieve the desired effect, but also produces more negative effects. Students’ learning of artistic content is a step-by-step process, and it is not possible to achieve significant results in a short period of time. The improvement of the management efficiency of public art education and the improvement of the quality of public art education require a gradual process. Even if the school's teaching conditions are good, but without good management, the quality of public art education would not be high; on the contrary, if the school’s teaching conditions are average, but the management is in place, and the teachers and students of the whole school work together, the quality of public art education can be greatly improved, and satisfactory results can be achieved. The level of management has a profound impact on the quality of public art education and the state of art teachers. According to the definition of dynamic range, the Dynamic Range Expansion factor (DRE) can be defined as the ratio of the maximum unsaturated signal P_VS_ obtained under the shortest exposure time to the maximum unsaturated signal P_max_ before dynamic range expansion^18,19^.

3 $$ {\text{DRE}} = \frac{{{\text{P}}_{{{\text{VS}}}} }}{{{\text{P}}_{{{\text{max}}}} }} = \frac{{{\text{T}}_{{\text{M}}} }}{{{\text{T}}_{{{\text{VS}}}} }} $$

For MOS transistors, the surface potential Ψ_s_ of the silicon body is mainly restricted by the voltage drop on the capacitor of the oxide layer under the condition that the gate voltage is $V_{FS}$, as shown in the following formula^20^.

4 $$ V_{g} = V_{FS} - \Psi_{S} $$

The management institutions of public art education in ordinary colleges and universities are roughly divided into the following types:

A separate institution: for example, such institutions as art education management centers or teaching and research offices are often independent and are specially responsible for managing the public art courses of the whole school and carrying out various art education activities.

Affiliated or affiliated with a certain institution: some schools would set up institutions for the management of public art education in colleges and universities in institutions such as the Party Committee Propaganda Department, the School Youth League Committee, and the Ministry of Education and Industry. Since some colleges and universities have their own art majors, the teaching and management of public art are often also the responsibility of teachers and institutions of art colleges^21^.

Organizations for mass organizations: some colleges and universities would set up organizations of mass organizations such as art troupes to be responsible for the management of the school’s student art troupes and the establishment of various public art courses in the school. Some art troupes are independent institutions in the school, while some art troupes are affiliated with institutions such as the Youth League Committee^22^.

### Diversified teaching modes of public art majors based on CMOS image sensor technology

Public art education in colleges and universities undertakes the responsibility of cultivating and improving students’ artistic aesthetic ability and cultural literacy, and disseminating China’s excellent national art culture and other countries’ excellent artistic achievements. The premise of the management of public art education in colleges and universities is to pay attention to and follow the laws of art education. In the management of public art education in colleges and universities, education must be used as a means and art as a medium to ensure the normative nature of art education. The formula definition of the absorption coefficient α is the ratio of the absorbed optical power ∆P after the light of the teaching equipment travels through the ∆z distance to the optical power P before starting this distance during public art teaching. The formula is expressed as follows^23^:

5 $$ {\upalpha }\left( {\uplambda } \right) = \frac{{\Delta {\text{P}}}}{{\Delta {\text{z*P}}}} $$

In public art teaching, the absorption length of teaching equipment is inversely proportional to the absorption coefficient, and the formula is as follows:

6 $$ L_{as} = \frac{1}{{{\upalpha }\left( {\uplambda } \right)}} $$

**Figure 2:**
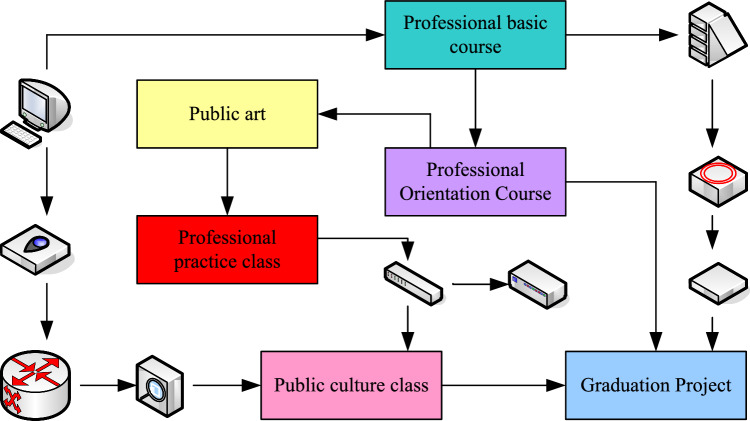
Teaching system of diverse public art courses.

In order to achieve the goal of public art education management in colleges and universities and improve the effect of public art education for college students, it is necessary to carry out a combination, control and adjustment of human, financial and material. The content of public art education management in colleges and universities includes the management of goals, mechanisms, principles, methods, processes, education, teachers and managers themselves and other aspects of public art education management in colleges and universities. The teaching system of diversified public art courses is shown in Fig. 2. The professional practice course is a comprehensive learning and exploration activity that students can carry out independently under the guidance of teachers. The courses of the professional practice course include project research, work creation, company project practice, social investigation, etc. Professional practice class is an opportunity for students to let go of their hands and feet to create boldly. Therefore, the proportion of professional practice class should be increased so that students can actually put into work in the unit to exercise^24^.

Incomplete charge transfer would not only cause smearing and noise in teaching multimedia video images, but also reduce the photoelectric conversion capability of teaching equipment during public art teaching, thereby reducing the dynamic range. Therefore, 4T active pixels with high charge transfer efficiency are the most basic requirements of large dynamic range CMOS image sensors, and the charge transfer transistors fabricated by standard technology cannot meet this requirement. Therefore, it is necessary to improve the device characteristics of photodiodes, transfer transistors and floating diffusion regions through process adjustments, so as to realize fast and complete charge transfer of teaching equipment during public art teaching. For extending the maximum unsaturated light intensity Pmax, the multiple exposure technology does not need to make any changes to the physical structure of the sensor. Only through simple timing adjustment, images of different exposure states can be obtained, and finally the images can be fused to obtain large dynamic range public art teaching projection images. However, for high-resolution public art teaching video shooting applications, the sensor is required to have a frame rate of 30 frames per second or even 60 frames per second. The adoption of multiple exposure technology requires the sensor to complete the acquisition and readout of all the different exposure state images in 1/30 of a second or 1/60 of a second. Therefore, although the application of multiple exposure technology is simple, it greatly increases the readout data rate and data volume of the sensor. In public art teaching, the sensitivity of teaching equipment is defined as the magnitude of the signal voltage value generated by the unit light intensity incident on the semiconductor surface per unit time. This quantity characterizes the sensitivity of the public art teaching pixel output signal to incident illumination. The formula for calculating the sensitivity of teaching equipment in public art teaching is as follows:

7 $$ {\text{S}} = \frac{{{\text{V}}_{{{\text{sig}}}} }}{{{\text{P}}_{{{\text{max}}}} \cdot {\text{t}}_{0} }} $$

The big difference between a CMOS image sensor and a CCD is that the CCD is serially read out, while the row and column gating circuit of the CMOS image sensor enables it to read some pixels randomly. The absorption of light in the silicon body is carried out according to a certain proportional coefficient, which is called the absorption coefficient. The absorption coefficient is defined as the ratio of the energy absorbed by light per unit distance in the silicon body to the energy of the light before that distance began. The larger the absorption coefficient, the faster the light is absorbed in the silicon body, and the faster the light decays from the surface to the interior. Conversely, the smaller the absorption coefficient, the slower the light is absorbed in the silicon body. According to the approximation of the depletion region, the expression of the depletion region width of the CMOS image sensor of the teaching equipment in public art teaching can be obtained:

8 $$ W_{D} = \frac{{2\varepsilon_{s} }}{q*V}\sqrt {\frac{{N_{A} N_{D} }}{{N_{A} + N_{D} }}} $$

The formula for the potential difference in the CMOS image sensor circuit of the teaching equipment during public art teaching is as follows:

9 $$ V_{bi} = \ln \frac{{kTN_{A} N_{D} }}{{n_{i} }} $$

Among them, k is Planck's constant and T is the absolute temperature. The formula for calculating quantum efficiency is as follows:

10 $$ {\upeta } = \frac{{\text{N}}}{{{\text{hv}}/{\text{P}}_{0} }} $$

Among them, P_0_ is the incident light power (unit is W) of the CMOS sensor of the teaching equipment during public art teaching, h is Planck's constant, and ν is the frequency of the incident light.

Ordinary colleges and universities should establish an art education management committee according to the situation of their own schools, or establish a permanent art education leading group. Supervisory leadership should be the principal or vice-principal. Then, according to the relevant policies of the Ministry of Education, the school art education development plan is formulated, and the curriculum is set according to the specific situation of the school. The teaching resources are coordinated, and the communication and coordination mechanism between various departments is established. Other departments or institutions coordinate to jointly promote the smooth implementation of arts education. At the end of the semester, the leading agency should organize relevant personnel to check and evaluate the effectiveness of art education work in each unit.

Therefore, although public art education has the content of improving professional ability, it also has the functions of cultivating morality, enhancing intelligence and strengthening physique. However, the improvement of professional ability, cultivation of moral education, intellectual education, physical education and other functions is not the main goal of art education for ordinary college students. The purpose of implementing art education is to expand students’ cultural vision and improve students’ aesthetic quality, as well as exercise their image thinking through art education, so as to help college students establish cultural awareness and enrich cultural heritage, as well as improve cultural literacy. It is the sacred mission of art education in ordinary colleges and universities to inspire the creativity of college students to cultivate their collaborative spirit. Therefore, the cultivation of students' aesthetic literacy should be regarded as the main purpose and task of art education in ordinary colleges and universities, and the unique role of art education in educating people in colleges and universities should be truly realized.

## Results of the construction of the teaching system for public art majors

Art education is an important part of education. Different scholars have different interpretations of its concept. It is necessary to train professionals who specialize in art work in the future in professional colleges and through professional teachers; art education in a broad category is art education for the public. Its purpose is to improve people's aesthetic ability and cultural literacy, and it is called "public art education" in the art education of colleges and universities.

Diversified public art teaching based on CMOS image sensor technology cannot limit its research to classroom teaching. Teaching is not isolated and closed, and the scope of public art learning should break through the field of traditional classrooms and be established in a wide range of social and cultural situations. Diversified public art teaching based on CMOS image sensor technology is a brand-new open and ecological teaching. The establishment of special institutions to be responsible for the daily work and curriculum management of public art education is an important link for public art education in colleges and universities. It is a clear requirement of the state for colleges and universities to have an independent public art education institution, which is especially responsible for implementing and carrying out the relevant regulations and tasks of the Ministry of Education and the Provincial Department of Education. According to the official website of each university, the website of the Department of Education, the official news release and the data of the leaders’ visits, a table is made according to the name of the public art education institution of each university, the time of establishment, whether it is independent, and whether there is a direct leadership of the school-level leaders. The standard of independence is set as follows: the institution is not affiliated or affiliated, and is independent from other departments of the school in terms of talent introduction, workload accounting, scientific research and professional title declaration. Some public art education institutions in colleges and universities are shown in Table 1.

**Table 1:** Some public art education institutions in colleges and universities.

Name of public art educational institution	Established (year)	Independent situation
Aesthetic education Teaching and research office	8	Yes
Music education center	10	Yes
Cultural and art education center	5	No
Art education Working committee	3	No
Name of public art educational institution	9	Yes

Most of the responses to “the most common teaching method used in public art classes” focused on the option “teaching”. That is to say, “lecture” is the most common teaching method used in public art courses, and although “discussion” is adopted, it is not used much in comparison. Although many teachers use multimedia courseware and video technology to teach, it is not fundamentally different from the traditional teaching methods of other non-art courses. Even if the method of discussion is adopted in the classroom, it is shallow and cannot go deep. Most of them are mere formalities, and students are rarely required to think positively, which makes students learn and accept knowledge completely passively in the whole teaching process. This virtually restricts students' thinking, resulting in students’ lack of initiative and enthusiasm in learning. In addition, 40% of the students say they like the teaching method of discussion, and 30% of the students like the teaching method of joint research. Through interviews, it is found that one of the reasons why many students are dissatisfied with the teaching of some art courses is that this teaching method based on lectures ignores the interaction between teachers and students and does not highlight the characteristics of art courses, as well as cannot arouse students’ interest (Table 2).

**Table 2:** Most common teaching methods used in public art classes.

Teaching form	Proportion (%)
Lecture	15
Discuss	40
Joint research	30
Other way	15

**Figure 3:**
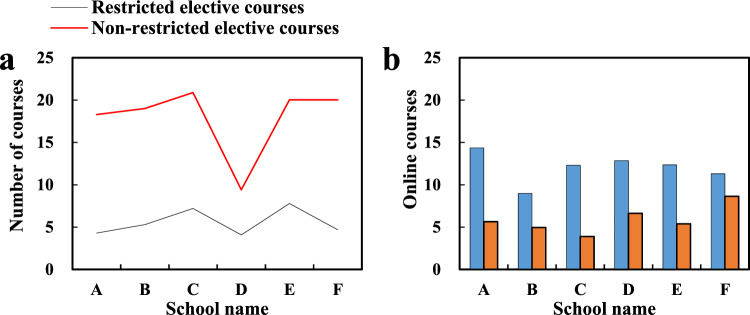
Course types and online course construction.

In the survey on restricted elective courses, 5 are 211 or 985 colleges and universities. There are still colleges and universities that are not fully opened, including 1 of the 4 comprehensive colleges and universities, 2 of the 6 science and engineering colleges, and 4 private colleges. In the survey of non-restricted elective courses, among the comprehensive colleges and universities, A and B have the largest number of courses, reaching 41 and 30. C University has 2, and D University has only 1 (“Physical Training and Etiquette”); among the science and engineering colleges and universities, D University of Technology has the largest number, offering 23 courses. Secondly, University E and University F also opened 14 and 10 courses respectively, and the other universities opened less than 10 courses respectively (the construction of limited elective courses and non-limited elective courses is shown in Fig. 3a).

For the construction of MOOCs, the largest number of them is that A University has opened 19 art MOOCs, and some universities have not yet opened them. Generally speaking, in the surveyed colleges and universities, half of the limited elective courses stipulated by the state are still incomplete; the number of non-restricted elective courses varies greatly among colleges and universities, and the types of courses offered are also unbalanced; in terms of online courses, various colleges and universities actively carry out the construction of MOOCs. Only some colleges and universities have not introduced online courses, but some colleges and universities have the phenomenon that half of the limited elective courses are set as MOOCs, or all rely on MOOCs (the construction of school online courses is shown in Fig. 3b).

**Figure 4:**
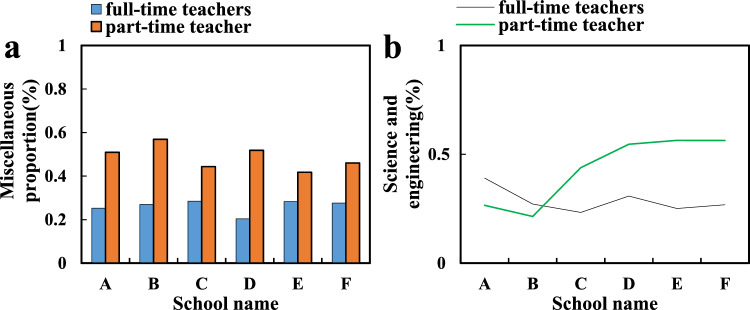
Faculty Types.

Among the universities surveyed this time, only 3 universities have teachers who teach public art courses, accounting for more than 0.15% of the total undergraduate students. The proportion of full-time teachers in the total number of art teachers in some universities is equal to or more than 0.5, and the rest of the schools have not reached the standard (the situation of teachers in comprehensive schools is shown in Fig. 4a). Among them, University A has relatively no shortage of professional art teachers due to the fact that it has various art departments such as the School of Music, the School of Fine Arts, the School of Letters, and the School of Journalism and Communication. More colleges and universities have not reached the national standard in terms of the number of teachers in public art education (the situation of teachers in science and engineering schools is shown in Fig. 4b).

**Figure 5:**
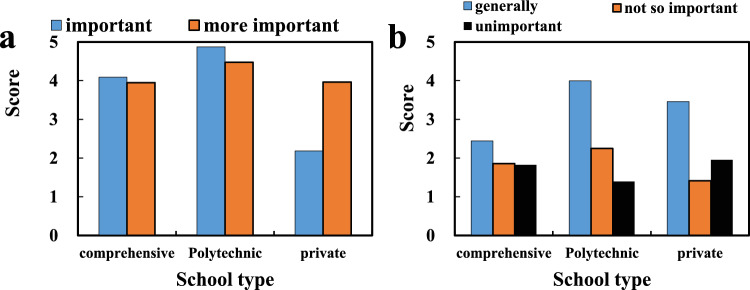
(**a**) Important, rather important. (**b**) Generally, not very important, not important.

Regarding the attitude of public art professional courses from the perspective of diversified public art based on CMOS image sensor technology, the importance of students to public art courses is divided into five levels (out of 5 points), which are: a full score of 5 means that public art courses are important (it is believed that public art education is of great significance to personal comprehensive quality, and public art courses are indispensable in colleges and universities), 4 points are considered more important (it is recognized that public art education is an important part of college education, and schools should offer relevant courses), 3 points are considered to be average (it is believed that public art education has a certain effect on the comprehensive quality of college students, but they may not choose public art courses), 2 points are considered not very important (the importance of public art courses is not recognized, and public art courses are considered optional), and 1 point is considered unimportant (public art education is considered useless, and schools do not need to offer public art courses at all). The results show that there are very few students who think that public art courses are not important or not very important, whether it is a comprehensive university, a science and technology university or a private university, and the average score given by the students is 4 points. It is close to the level of “relatively important” (important, relatively important, as shown in Fig. 5a), indicating that most of the interviewed students agree that public art education is an important part of college education, and schools should offer relevant courses (generally, less important, unimportant as shown in Fig. 5b).

Students can choose courses only by checking the timetable published by the Academic Affairs Office, but they are not clear about the course positioning, specific content, teaching methods, etc. In this investigation and visit, some students even think that they could not learn public art courses without the foundation of professional learning such as vocal music and instrumental music, and they do not choose public art courses because they are worried that they would not understand. For colleges and universities that have carried out public art education for only a few years, the tradition of emphasizing majors and ignoring art is still in study. Some colleges and universities are still unwilling to increase investment in public art education. There are many situations in which public art education teachers are completely replaced by professional art teachers. In addition, there is no good policy to attract highly educated and high-level teachers, and there is not enough administrative staff, which also greatly restricts the development of public art courses.

The goal of public art education in colleges and universities is to cultivate the innovative thinking ability of college students and improve artistic accomplishment, as well as shape excellent morality. When learners feel relaxed and interesting in the process of participating in public art education, the learners’ satisfaction with the course would be higher. With the gradual deepening of the course study, the learners’ perception of the course learning experience is dominant. The quality of the course is high. The content is interesting and relaxed, and the knowledge demands are satisfied, which produces a sense of satisfaction and urges the learners to continue the course study; finally, learners who have completed the course unknowingly, are used to or love the course, and may choose to continue learning because they are reluctant to give up some learning resources and interpersonal relationships. At present, with the continuous advancement of education informatization and public art education in colleges and universities, more and more learners are participating in public art education in colleges and universities, and it is of great academic and practical value to study the satisfaction of these learners.

**Figure 6:**
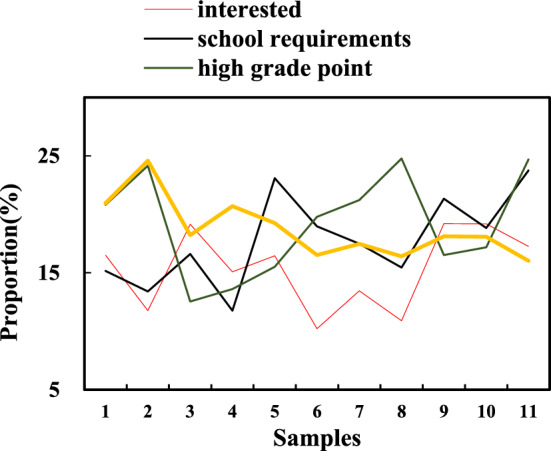
Reasons for choosing a public art program.

Among the respondents to this questionnaire survey, more than 80% (82.26%) of the students indicated that they had taken public art courses. It shows that on the one hand, public art courses are more popular in colleges and universities, and most colleges and universities offer them. On the other hand, the enthusiasm of college students is also relatively high, so many respondents would attend classes. For the college students who participated in the art public course, the main reason for choosing the course is their interest in the art subject (25%), indicating that their own interest is the main starting point of the college students. This is followed by school mandates, which are chosen by nearly a quarter (22%) of students. This shows that some rules and regulations of the school would obviously affect the students’ course choices. For college students who have not taken the course, the main reason for not taking the course is that the school does not have strict requirements (37%), followed by being tired of various professional courses (19%). The main responsibility of college students is indeed to learn their own professional courses well. However, art education also plays an important role in cultivating the comprehensive quality of college students. Therefore, schools cannot despise public art education, and students themselves cannot ignore the importance of art education due to the lack of compulsory requirements in schools and the heavy workload of professional courses. Instead, a certain number of art courses should be taken in moderation as permitted. The reasons for choosing public art courses are shown in Fig. 6.

**Figure 7:**
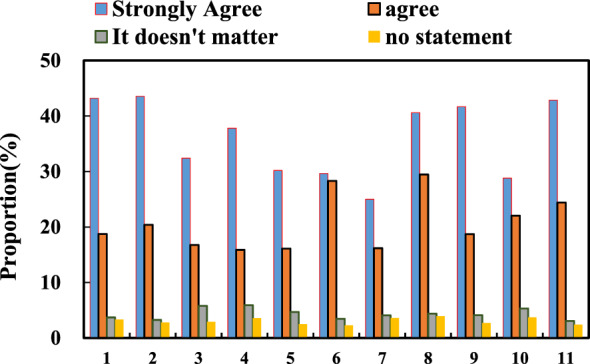
Students’ evaluation of improved artistic literacy.

Everyone has reached a consensus that the public art major courses from the perspective of diversified public art based on CMOS image sensor technology play an important role in improving students' artistic literacy and promoting students' all-round development. This can also be proved from the survey of students and teachers in some colleges and universities. Among the students surveyed, 85.2% believe that it is beneficial for personal development to participate in public art courses from the perspective of diverse public art based on CMOS image sensor technology, although the emphasis is different. 50% of the students have a very positive attitude towards the establishment of public art professional courses from the perspective of diversified public art based on CMOS image sensor technology. 30% of the students agree with this, and 80% of the students agree, and only 10% of the students have an indifferent attitude. 6% of the students disapprove of the establishment of public art professional courses based on the diversified public art perspectives based on CMOS image sensor technology, and 4% have no opinion. 81.3% of students believe that the main purpose of participating in public art majors from the perspective of diverse public art based on CMOS image sensor technology is to improve artistic literacy and increase knowledge, as well as enrich their spare time. Only 10.8% of students are for credit, and 7.9% do not answer this question. The students’ evaluation of the improvement of artistic literacy is shown in Fig. 7.

**Figure 8:**
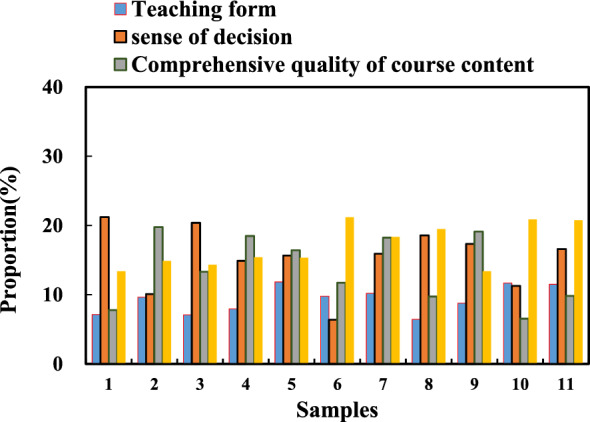
Learner Satisfaction Distribution.

15% of the students expressed “satisfaction” with the CMOS teaching form, indicating that the teaching form in the CMOS image sensor mode of public art education in colleges and universities has been well recognized. 10% of students were “satisfied” with the decision to participate in the course, and 30% were “very satisfied” with the decision to participate in the course. 51% of the students expressed “satisfaction” with the operation of each module of the CMOS image sensor platform in the course, indicating that the CMOS image sensor teaching platform has a good user experience, which is conducive to promoting learners’ platform experience in the learning process. 63% of the students expressed “satisfaction” with the teachers’ comprehensive quality ability, indicating that the relevant teachers have a high level in the aspects of subject literacy, information technology, and teaching ability in the process of trying to informatize public art courses. 56% of the students expressed “satisfaction” with the overall quality of the course content, indicating that the video and document content of the public art CMOS teaching course are reasonably designed, easy to understand, and attractive; the media presentation forms such as pictures, animations, sounds, and cases are rich. 56% of the students expressed “satisfaction” with the whole teaching activity, indicating that the discussion, communication, evaluation, assessment and other links in the diversified public art CMOS teaching are rich and complete, which is more conducive to the internalization of knowledge. 38% of the students expressed “satisfaction” with the whole learning experience, and 46% expressed “very satisfaction”. It shows that the construction of public art education courses from the perspective of diversified public art based on CMOS image sensor technology has achieved good teaching results as a whole. The distribution of learner satisfaction is shown in Fig. 8.

## Conclusion

The relationship between diversified public art and public art education in ordinary colleges and universities is getting closer and closer, and there are many possibilities for its future development. This paper used CMOS image sensor technology to simulate various three-dimensional spaces, including abstract spaces such as historical space and art space. The use of CMOS image sensor technology creates a virtual public art teaching platform that allows students to see, hear, and even touch these artworks. The use of CMOS image sensor technology can establish a virtual art operation space and expand students’ hands-on ability. Public art education from the perspective of diversified public art based on CMOS image sensor technology can make public art education in colleges and universities more interesting, and play an important role in promoting public art education in colleges and universities. The digital interactive courseware produced in this paper has a small amount of information and cannot carry too much teaching content. Therefore, although it can be proved that the assisted teaching of diversified public art based on CMOS image sensor technology can improve students’ interest in learning, it is impossible to deeply explore how helpful the diversified public art based on CMOS image sensor technology can be to students’ learning. In the future, researchers in public art education can find out deeper into related technologies to improve digital interaction design. This will lead to the creation of high-quality digital interaction courseware and the acquisition of valuable data and information through practical application. Ultimately, this will enhance the persuasiveness of research in this field.

## Data Availability

Data can be provided upon reasonable request by contacting the corresponding author.
